# Development and characterization of short glass fiber reinforced-waste plastic composite filaments for 3D printing applications

**DOI:** 10.1016/j.heliyon.2023.e22333

**Published:** 2023-11-17

**Authors:** Dame Ayane Tolcha, Dereje Engida Woldemichael

**Affiliations:** Addis Ababa Science and Technology University, College of Engineering, Mechanical Engineering Department, Addis Ababa, Ethiopia

**Keywords:** 3D printing, Composite filament, Recycled polyethylene terephthalate, Recycled high-density polyethylene, Short glass fiber, Waste plastic

## Abstract

In the present study, a thermoplastic-reinforced composite filament for 3D printing applications was created by mixing short glass fibers (SGF) with the thermoplastic matrix made from plastic waste. Short glass fiber-reinforced recycled high-density polyethylene (rHDPE) and recycled polyethylene terephthalate (rPET) blend thermoplastic composite filaments were first prepared by a plastic extrusion machine. The produced filaments' physical, mechanical, and thermal properties were investigated. After achieving the desired filament, a 3D printing technology called material extrusion was utilized to create 3D sample parts. The mechanical properties of each printed sample were analyzed following ASTM standards, and their morphological structures were also studied at various weight percentages (pure rHDPE, rHDPE/rPET (75/25 %), rHDPE/rPET reinforced with 15 % SGF, and rHDPE/rPET reinforced with 30 % SGF wt%). From the study, a 52 % increase in tensile strength, 32 % in Young's modulus, and a 50 % reduction in elongation with the addition of 30 wt% of SGF to rHDPE/rPET were noted compared to pure rHDPE. Generally, 3D printable composite filaments can be developed through the incorporation of SGF into plastic waste. This has tremendous advantages for solving environmental pollution and achieving sustainable development.

## 1 Introduction

The 2030 Agenda for Sustainable Development Goals of the United Nations challenges us to create sustainable infrastructure and services to overcome high energy consumption, solid waste generation, water resource depletion, and greenhouse gas emissions in many industries [1]. Sustainable development is established to preserve the resources available now to ensure the same benefits and opportunities are available in the future. This concept of sustainability has prompted the need to minimize or reduce waste, popularize the idea of reuse, and encourage recycling products after they are disposed of [2]. According to statistical extrapolation, 12,000 million metric tons of plastic will accumulate in landfills by 2050 if the current rate of plastic waste generation continues [3].

Due to rising demand in developing nations such as Ethiopia, global plastic production has expanded dramatically over the past few decades. The amount of plastic consumed per capita in Ethiopia has increased by roughly 13.1 % per year over the past few years, rising from 0.6 kg in 2007 to 2.6 kg in 2018, and is predicted to reach 3.8 kg in 2022 [4]. So, plastic waste management options are currently unable to handle the waste plastic produced, and the methods for disposing of plastic garbage have transformed into valuable products. Therefore, using waste plastic materials in modern manufacturing techniques is essential to achieving sustainable development and increasing the recycling rate of waste plastic. Today, 3D printing technology is a fast-growing manufacturing technique employed in various industries. Inducing plastic waste materials in 3D printing has the most significant potential as a solution for waste plastic impacts and can ensure sustainable development [[5].

Nowadays, additive manufacturing (AM), a.k.a. 3D printing, is a widely used method of rapid prototyping and has recently gained a lot of attention and attracted the interest of everyone, from entrepreneurs to at-home hobbyists [6]. It is an upcoming technology in the manufacturing industry that entails the use of computer software (CAD) to command the machines to create the desired material layer by layer. AM has grown popular because of its potential and benefits such as design freedom and short production periods [7,8]. There are various classifications of AM processes, including photopolymerization, material jetting, binder jetting, directed energy deposition, powder bed fusion, sheet lamination, and fused deposition modeling or material extrusion [9,10]. Fused deposition modeling (FDM) is one of the commonly used AM techniques due to its low cost, minimal waste, and ease of material change. It was initially developed for polymeric materials, waxes, and paper laminates and later expanded to composites, metals, and ceramics [11]. Thermoplastic polymers are the most often used materials for the FDM process due to their relatively low cost and melting temperatures. They are the most widely adopted materials and are frequently created in the shape of a long wire wrapped on a spool, sometimes known as a filament. Thermoplastic materials such as polyurethane (TPU), acrylonitrile butadiene styrene (ABS), polyethylene terephthalate (PET), polylactic acid (PLA), PEEK (polyether ether ketone), and nylon are some of the materials that can be used [[12], [13], [14], [15], [16]]. The popularity of desktop 3-D printers is increasing the demand for recycled 3-D printer filament, which is used to reduce distribution costs. Therefore, the manufacturer can decide to reuse the plastic waste by turning it into filament and manufacturing the desired objects. It might present challenges with losses in mechanical properties that do not restrain the use of recycled filament for a model object [17]. To solve this problem, reinforcing materials such as fibers are included in the polymer matrix to generate a composite structure with superior mechanical properties. Additive manufacturing of short fiber-reinforced polymer composites is perhaps the most common reinforcement used in 3D printing. Short-fiber composites are desirable because of their ease of fabrication, low cost, and excellent mechanical qualities. As a result, they are attractive methods in many AM applications [18,19].

Short fibers, such as glass and carbon fibers, are commonly employed as reinforcements in 3D printing to improve the mechanical properties of polymer composites. Typical of SFRTC, a thermoplastic polymer matrix contains relatively small fibers of varying lengths that are randomly distributed or inadequately aligned. Additive manufacturing technologies, such as fused filament fabrication (FFF), are becoming popular with short fiber-reinforced thermoplastic composites [20,21]. Extrusion is the widely utilized processing method for producing components with SFRTCs. The filaments are made by combining polymer pellets and fiber in a blender and extruding the mixture to make the filament. The technique of heating and melting a polymer or fiber blend to a working temperature and forcing the mixture through a small hole or die of the required shape is known as polymer composite extrusion [22]. Many studies have shown that increasing the amount of fiber results in an increase in the mechanical properties of the material, particularly in stiffness and tensile strength. Short carbon fibers added to the ABS polymer, which Tekinelp et al. discovered, increased the tensile strength and Young's modulus by 115 and 700 %, respectively [22]. Ning et al. investigated the effect of fiber content on the mechanical properties of an FDM-printed ABS/carbon fiber composite and noticed an increase in mechanical properties [23].

Thermoplastic materials and glass fiber combinations can achieve good mechanical properties in printed parts. However, short-glass-reinforced waste thermoplastic composite is a novel feedstock with lower material costs for 3D printing applications. This work developed SGF-reinforced rHDPE/rPET blend composite filaments to use for the material extrusion of the 3D printing process. First, a composite filament was produced or manufactured. Physical properties (consistency, tolerance, and roundness), water absorption, differential scanning calorimetry analysis, and tensile testing are conducted to investigate the properties of proposed filaments with varying glass fiber content (fiber weight ratio). The mechanical and morphological properties of 3D-printed specimens are also studied. Finally, the studied properties have been compared to those of pure recycled rHDPE and commercial filament.

**Table:** Nomenclature

rHDPE	recycled high-density polyethylene
rPET	recycled polyethylene terephthalate
FDM	Fused deposition modelling
SFRTC	Short fiber reinforced thermoplastic composite
DSC	Differential scanning calorimeter
SEM	Scanning electron microscopy

## 2 Materials and methods

### 2.1 Raw materials

**Fig. 1:**
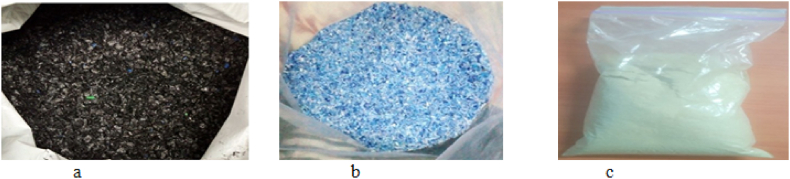
Photograph of as received (a) recycled HDPE flakes, (b) recycled PET flakes, and (c) short glass fiber.

The recycled HDPE used for this research, shown in Fig. 1a, and recycled PET in flake form (PET-flake) Fig. 1b, supplied by COBA Company (Addis Ababa) and Ethio-Plastic company (Addis Ababa), were used as a major and minor matrix, respectively. Short glass fiber was used as an additive to reinforce recycled plastic. Fig. 1c is the glass fiber used in this study, prepared by filtering with a mesh size 160 μm.

### 2.2 Filament fabrication

After the granulating process, a Contherm Thermotech oven was utilized to dry the raw material and remove moisture from it. HDPE/PET blend composition was prepared as shown in (Table 1) adopted from Chen et al. [24] due to its ease of fabrication and cost-effectiveness.

**Table 1:** Preparation of material composition.

Materials	Weight composition
rHDPE	100 %wt
rHDPE/rPET	75/25 %wt
rHDPE/rPET/GF	64/21/15 %wt
rHDPE/rPET/GF	53/17/30 %wt

The prepared materials were compounded and extruded using a single-screw extrusion machine for ease of extrusion and to reduce cost. Then, the rPET flakes and rHDPE recovery composite with glass fiber were extruded into a 1.75-mm-diameter filament in the single screw extruder machine (type Y100). The extruder machine has four temperature zones: feeding, barrel 1, barrel 2, and nozzle.

Four parameters were considered in this work, namely: extrusion temperature, cooling method, spooler speed, and screw speed. By using these parameters, the extrusion process was performed according to the Taguchi approach to achieve the best filament diameter. In the present work, two levels and four factors are identified; therefore, L8 has been selected. By using the parameters that had been determined, the extrusion process of rHDPE was performed according to the design of experiments (DOE) that has been made in the form of Table 2. For each extrusion process, filament diameter was measured using a digital vernier caliper.

**Table 2:** Prepared design parameters for the rHDPE extrusion process.

			Parameter	
Experiment	Extrusion temperature	Extrusion speed (rpm)	Cooling system	Spooler speed (Rpm)
1	180-190-200-210	20	Water cooling	2
2	180-190-200-210	20	Air cooling	3
3	180-190-200-210	30	Water cooling	3
4	180-190-200-210	30	Air cooling	2
5	190-200-210-220	20	Water cooling	3
6	190-200-210-220	20	Air cooling	2
7	190-200-210-220	30	Water cooling	2
8	190-200-210-220	30	Air cooling	3

Again, the rHDPE/rPET (75/25 %wt), rHDPE/rPET with glass fiber weight ratios were prepared according to Chen et al.'s [25]study, used as starting points for the processing extrusion temperature, and processed according to Table 3.

**Table 3:** Prepared design parameters for rHDPE/rPET, 15 %GF and 30 %GF extrusion process.

			Parameter	
Experiment	Extrusion temperature	Extrusion speed (rpm)	Cooling system	Spooler speed (rpm)
1	230-240-235-245	20	Water cooling	2
2	230-240-235-245	20	Air cooling	3
3	230-240-235-245	30	Water cooling	3
4	230-240-235-245	30	Air cooling	2
5	240-250-245-255	20	Water cooling	3
6	240-250-245-255	20	Air cooling	2
7	240-250-245-255	30	Water cooling	2
8	240-250-245-255	30	Air cooling	3

**Fig. 2:**
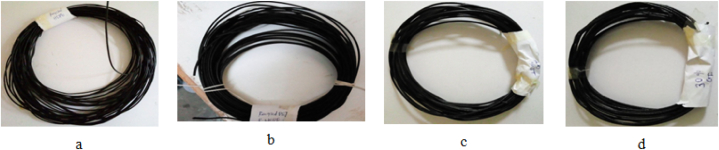
Photograph of filament developed a) rHDPE, b) rHDPE/rPET (75/25 wt%), c) rHDPE/rPET with 15 % GF d) rHDPE/rPET with 30 % GF (75/25 wt%).

From the design of experiments prepared for the extrusion process, filaments shown in Fig. 2 were fabricated with extrusion temperatures of 180–190–200–210 °C, an extrusion speed of 30 rpm, a water cooling method, and a spooler speed of 2 rpm for rHDPE (Fig. 2a) were used to develop an optimal filament diameter of 1.74 mm, with a +0.01 mm difference and a 0.0016 standard deviation compared to 1.75 mm commercial filament. Again, extrusion temperature (240–245–250–255 °C), extrusion speed (20 rpm), water cooling method, and spooler speed (3 rpm) of design experiments are optimal parameters for composite filament rHDPE/rPET (75/25) (Fig. 2b); 15 % (Fig. 2c) and 30 % (Fig. 2d) reinforced short glass fibers were similar with commercial filament (1.75 mm).

### 2.3 Filament characterization

The fabricated filaments in Fig. 2 are characterized according to ISO/ASTM 52903-2. The main properties are the physical properties (filament diameter tolerance, roundness, consistency, water absorption), mechanical properties, thermal properties (differential calorimeter analysis), and morphology properties.

#### 2.3.1 Physical properties (filament consistency, diameter tolerance, and roundness)

##### 2.3.1.1 Filament consistency

Filament consistency influences the flow rate in the course of extrusion, resulting in poor surface quality, extruder jams, irregular gaps between individual extrusions, and excessive overlap, leading to failed 3D prints. From a 2.00 ± 0.01 m sample of extruded filament, consistency was evaluated by measuring filament diameter and mass per unit length. The filament length was divided into 20 sections of 0.100 ± 0.003 m part GB/T 37643. A digital micrometer measured the diameter at the section's midway point. The mass of each divided section was measured using a digital scale (0.0001 g) and compared using mass per unit length (0.001 g/100 mm).

##### 2.3.1.2 Filament diameter tolerance

Tolerance is a measurement of the diameter variation. Usually, filament should maintain a consistent diameter across the entire spool, but due to manufacturing processes, tolerances are always allowed depending on the size of the filament generated. However, because inconsistent extrusion leads to varying diameters, special attention should be paid to extrusion [25]. The diameter of the filament was measured with a digital micrometer at different points for each position, with the average value provided. By subtracting the formulated diameter (1.75 mm) from each average value, the diameter tolerance is calculated according to the GB/T 37643-2019 standard.

##### 2.3.1.3 Filament roundness

The uniformity of the filament during the manufacturing process is critical throughout the spool's length. Because of the grasping and rotating winding, the filament's diameter compressed when it came into contact with the wheels. According to the GB/T 37643-2019 standard, the filament roundness was estimated by subtracting the maximum diameter from the minimum diameter, and the permissible roundness tolerance was 0.05 mm.

#### 2.3.2 Water absorption and diameter swelling test

The water absorption data is crucial to understand how polymeric materials behave during processing (such as when printing model objects) and in wet or humid settings. Following ASTM D570, four specimens per material (length = 50 ± 0.1 mm) were prepared and submerged to water for a predetermined time to calculate the water absorption rate. The specimens that underwent preconditioning were weighted ($W_{0}$) using an electronic weight balance. Later, the samples were placed in distilled water for 24 h at room temperature. Then, the body of the piece was wiped off with a dry cloth, the weight ($W_{i}$) was recorded, and the specimens were re-immersed in the water. The weight readings were repeated and recorded every 24 h for seven days. The difference between the substantially saturated and conditioned weights is used to calculate the amount of water absorbed while substantially saturated. Equation 1 is used to compute the percentage change in weight during the immersion.

(1) $$increase\;in\;weight\left(weight\;\%\right)=\frac{\left(W_{i}-W_{0}\right)}{W_{o}}\times 100$$

The swelling diameter of the filament was determined by measuring the diameter of each specimen before (*D*_*0*_) and after (*D*_*i*_) the water immersion test, according to Equation (2).

(2) $$Diameter\;swelling\left(\%\right)=\frac{\left(D_{i}-D_{0}\right)}{D_{o}}\times 100$$

#### 2.3.3 Thermal analysis

The thermal analysis of filaments was utilized by differential scanning calorimetry (DSC). It is used to study what happens to polymers or samples upon heating, and it is used to study the thermal transitions of a polymer or sample (the changes that occur on heating), such as the glass transition and melting temperature. The samples were taken from filaments; their masses were 17 mg according to ASTM D3418 and carried out using an SKZ1052B differential scanning calorimeter (SKZ Industrial Co., limited). Samples were heated from 30 °C to 350 °C with a 10 kJ/min heating rate. By using Equation (3)**,** the crystallinity value for melting (*Xm*) was calculated [26].

(3) $$X_{c}\left(\%\right)=\frac{\Delta H_{m}}{f.\Delta H^{o}}\times 100\%$$

where:

f = weight fraction of matrix in the composite

*X*_*c*_*(%)* = degree of crystallinity

$\Delta H_{m}$ = specific enthalpy of melting obtained in DSC

$\Delta H^{o}$ = specific enthalpy of melting for 100 % crystalline polyethylene

The melting enthalpy ($\Delta H^{o}$) of 100 % crystalline matrix is obtained from literature, 293 J/g [27]**.**

#### 2.3.4 Mechanical properties of filament (tensile tests)

Tensile tests of the developed filament were carried out using the ASTM D638 standard. It is conducted on a DEVOTRANS machine and compared with reference samples or commercial filament (PLA). This test was used to determine the maximum strength, breaking strength, breaking elongation, elastic materials, yield stress, and elastic modulus. The following parameters were used: a constant displacement rate of 200 mm/min, a stress modulus velocity rate of 250 mm/min, a preload of 5 N, and a preload speed of 50 mm/min.

### 2.4 Printability of the proposed filament

All additive manufacturing parts must begin with a software model that depicts the external geometry. A 3D CAD modelling software, SolidWorks, was used to generate the geometry of the test specimens and save it in a stereolithography file (STL). Because of its semi-crystalline nature, HDPE as a feedstock material requires special preparation or a hotbed. Lack of adhesion is the main problem that faces high-density polyethylene. This poor adhesion has been problematic owing to massive shrinkage, voiding, and warpage problems. The filament feed was at a higher extrusion temperature to avoid issues with semi-crystalline plastic**.** Hence it requires to have a relatively high nozzle temperature in the range of 230 °C and 260 °C. The bed temperature's upper limit was between 127 °C and 130 °C [15,28]**.** Higher bed temperatures promoted more adhesion between the HDPE and the printing surface. So, several printing parameters were determined according to research by Schirmeister et al. on 3D printing of high-density polyethylene by fused filament fabrication [15]**,** and several observations were gathered from the preliminary testing or trial and error of the printing parameters to minimize these unwanted characteristics. Again, printing parameters such as filling pattern and filling density are done according to a study on the effect of fused deposition modeling process parameters on the mechanical properties of 3D printed parts by Chadha et al. [29]. Unlike other printing patterns, the triangle pattern contains lines printed in triaxial directions. Due to this, the strength in each direction will be uniform and resistant to outside forces. Table 4 shows the optimal printing parameters for the 3D-printed test specimens.

**Table 4:** 3D Printing parameters.

Parameters	Values
Nozzle temperature	250
Filling density	100 %
Printing head speed	30 m/s
Raster angle	0
Filling pattern	Triangular
Bed temperature	110
Layer height	0.2

**Fig. 3:**
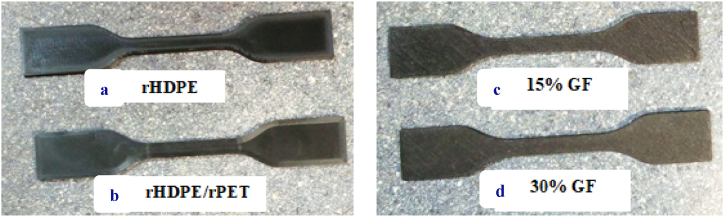
Photograph of a tensile test specimen (a) rHDPE, (b) rHDPE/rPET (75/25) blend, (c) 15 % GF, (d) 30 % GF composite filament.

When all parameters filled, we can create a slice, save it to a disk, and send it to the 3D printer. The Ultimaker printer uses CURA software as a slicer for SOLIDWORKS drawings to create objects, Fig. 3.

#### 2.4.1 Tensile test printed specimen

Test specimens in Fig. 3 are prepared in accordance with ASTM D638 Type IV and tested for tensile strength on a universal testing machine (UTM). Tensile property measurement was carried out on the following specification: AutoGraph UTM has a grip attachment distance of 33 mm and applies a load of 5.0 kN at a constant speed of 5 mm per minute. Tensile testing is a mechanical property test commonly used to obtain information about a material's ultimate tensile strength, stress, strain, elongation, and so on.

#### 2.4.2 Scanning electron microscopy measurements

Fractures of failed tensile composite specimens were observed using a JCM-6000Plus benchtop SEM (JEOL) . Samples were prepared by gold sputtering for 1 min at 20 mA to obtain a good-quality SEM image and good conductivity. The fracture surface, morphology, and glass fiber distribution within the rHDPE and rPET matrix were investigated.

## 3 Results and discussions

### 3.1 Filament characterization results

The developed filaments are characterized for their physical properties (filament diameter tolerance, roundness, consistency, water absorption), mechanical (tensile testing) properties, thermal properties (differential scanning calorimeter), and morphology (SEM) in the following sub-sections.

#### 3.1.1 Filament's diameter consistency, tolerance, and roundness results

##### 3.1.1.1 Filament consistency

The diameter of the produced filaments was measured using a digital micrometer with a tolerance of 0.001. The average filament diameter was measured at 20 consecutive locations along the filament's 2 m length. The average diameter of the filament produced can be calculated by taking the mean of the individual measurements shown in Equation 4, and we get 1.74054 mm, 1.7632 mm, 1.7654 mm, and 1.7702 mm for rHDPE, rHDPE/rPET (75/25), 15 % GF, and 30 % GF, respectively, in diameter.

$$\bar{x}=\frac{\sum_{i}^{n}x_{i}}{n}\left(4\right)$$

##### 3.1.1.2 Diameter tolerance

The diameter of the filament was measured with a digital micrometer at three different locations for each position, with the average value provided. By subtracting the formulated diameter (1.75 mm) from each average value, the diameter tolerance was calculated by Equation 5.

(5) Averagediametertolerance=averagediameter−1.75mm

Then, we obtained the value of diameter tolerance as 0.0095, 0.0132, 0.0154, and 0.0202 for rHDPE, rHDPE/rPET (75/25), 15 % GF, and 30%GF respectively, which is less than 0.03.

##### 3.1.1.3 Filament diameter roundness

According to the GB/T 37643-2019 standard, the filament roundness was estimated by subtracting the maximum diameter from the minimum diameter sampled at three positions by a digital micrometer, and the permissible roundness tolerance is 0.05 m. By using Equation (6), the maximum filament roundness of rHDPE, mixed rHDPE and rPET, rHDPE/rPET/15%GF, and rHDPE/rPET/30%GF are 0.053, 0.36, 0.37, and 0.42, respectively, which are within the range of recommended standard filament.

(6) FilamentDiaRoundness=maximumdiameter–minimumdiameter

**Fig. 4:**
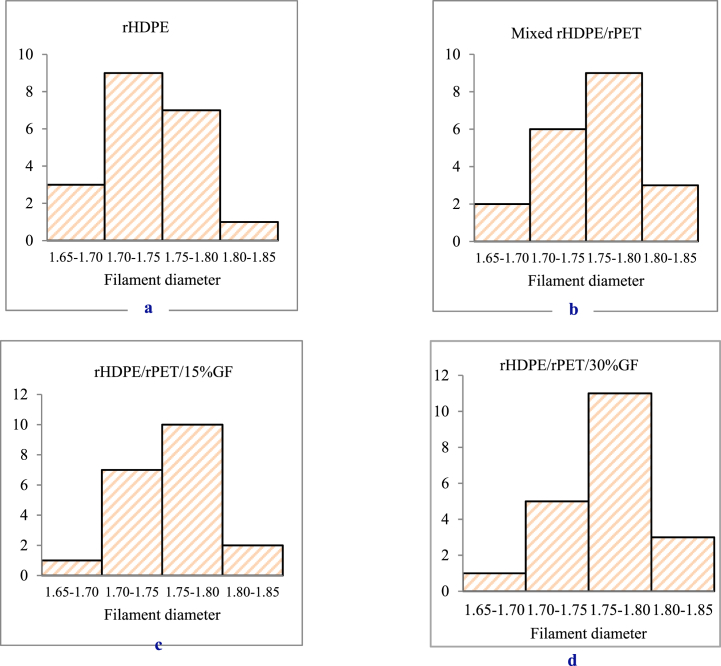
Frequency distribution of filament diameter (a) rHDPE, (b) rHDPE/rPET (75/25) blend, (c) 15 % GF, (d) 30 % GF composite filament.

The majority of the filament distribution, as shown in Fig. 4, falls between 1.70 and 1.75 mm for the recycled HDPE filament and rHDPE/rPET (75/25 wt%), rHDPE/rPET with 15 % glass fiber (rHDPE/rPET/15%GF), and with 30 % glass fiber (rHDPE/rPET/30GF); the filament diameter distribution is between 1.75 and 1.80 mm, which is within the range of standard filaments.

#### 3.1.2 Water absorption and swelling diameter

**Fig. 5:**
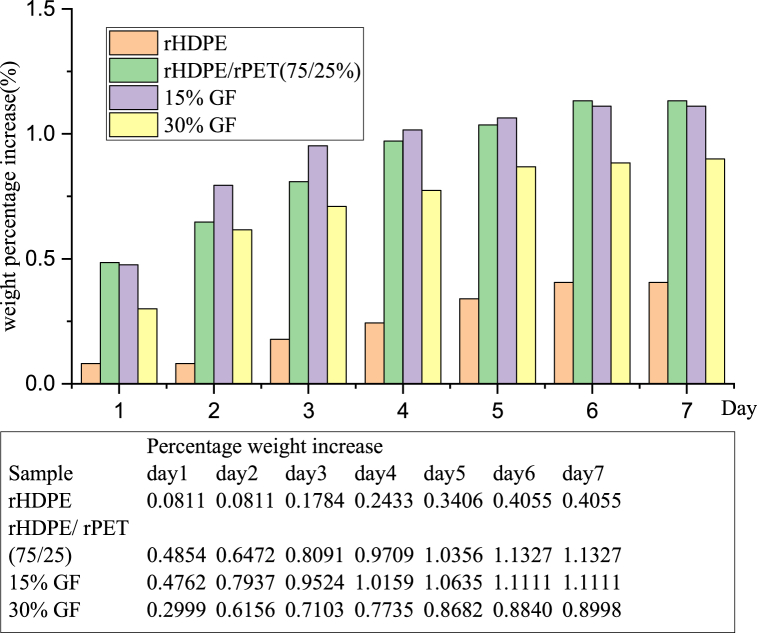
Percentage weight increase of filament.

**Fig. 6:**
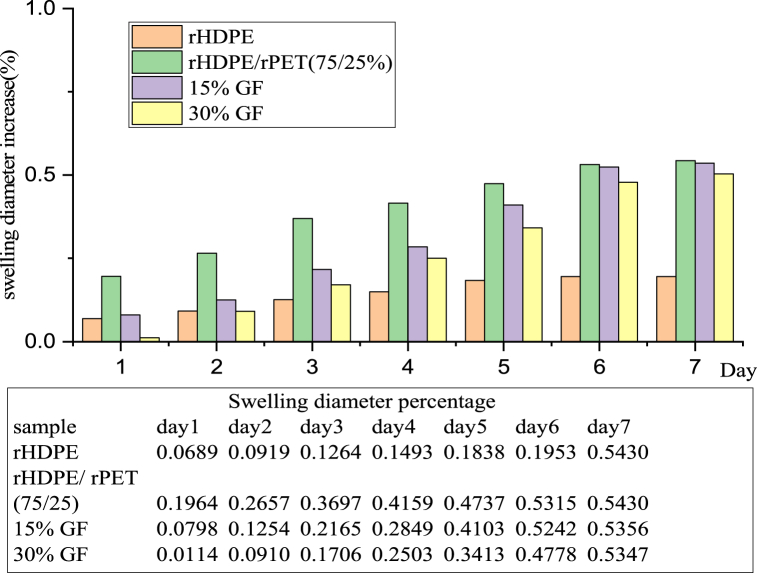
Percentage diameter increase of filament.

The conditioned samples were immersed in distilled water, and their weight was measured every 24 h for seven days after wiping away all moisture content with a dry cloth. The filament's weight percentage increase was determined using equation (1); the rHDPE/rPET blend composite has slightly higher water absorption and a higher swelling diameter due to the glass fiber content, as shown in Fig. 5, Fig. 6. The water absorption of rHDPE/rPET (75/25 %) is slightly higher than that of other produced filaments. Compared to rHDPE/rPET (75/25 %) and 15 % GF-reinforced composites, 30 % GF-reinforced blend plastic composites have the lowest water uptake of 50 %. This shows the incorporation of 30 % glass fiber (GF) into the thermoplastic matrix, which aids in reducing water absorption and improving fiber-matrix interfacial bonding. As a result, incorporating glass fiber into the rHDPE/rPET matrix improves its mechanical properties while lowering the moisture intake rate of the composite filaments. This conclusion is further supported by the work of Oladele et al. who established that the hybrid composites' moisture intake is decreased when glass fiber is added to a polypropylene matrix [30].

The swelling diameter percentage of the filament was determined by measuring the diameter of each specimen before (*D*_*0*_) and after the (*D*_*i*_) water immersion test, according to Equation (2). The statistical analysis showed that all of the specimens with different compositions had different swelling diameters. The increase in swelling specimen diameter is shown in Fig. 6. The results varied from sample to sample, and rHDPE/rPET (75/25 %) showed some slightly higher than others due to the filament's pores and the specimen's poor interaction matrix.

#### 3.1.3 Tensile test of manufactured filament

**Fig. 7:**
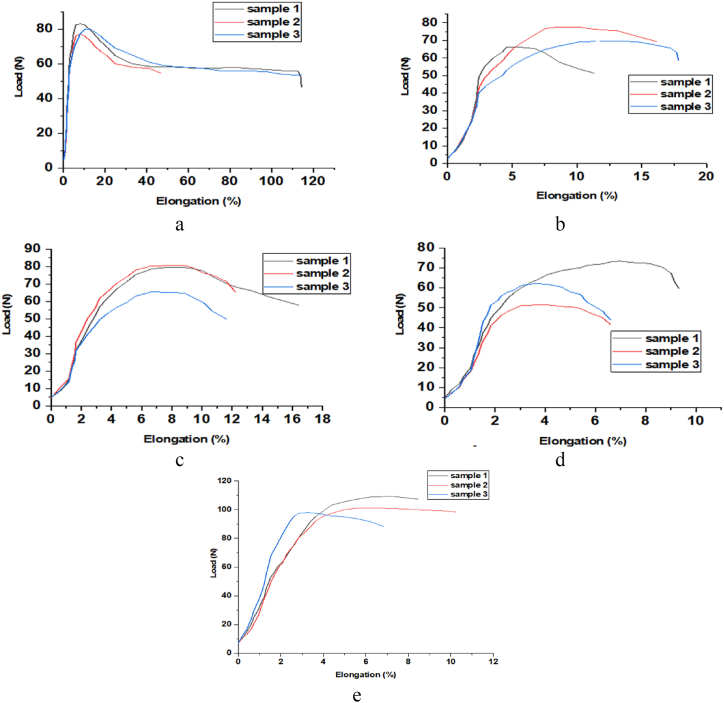
Force versus elongation of (a) rHDPE, (b) rHDPE/rPET (75/25) blend, (c) 15 % GF, (d) 30 % GF composite filament and (e) PLA filament.

The mechanical properties of prepared filament were compared with the most widely used commercial PLA filament for rapid prototyping to evaluate the potential application of recycled HDPE, mixed rHDPE/rPET (75/25 %), 15 % GF, and 30 % GF reinforced filament for 3D printing filament. Samples of each filament and commercial PLA filament were subjected to tensile tests. The load-elongation curve is generated by the DEVOTRANS tensile test of each filament manufactured, and commercial PLA filament is shown in Fig. 7 where Fig. 7a is for rHDPE, Fig. 7b is for rHDPE/rPET (75/25) blend, Fig. 7c is for 15 % GF, Fig. 7d is for 30 % GF composite filament and Fig. 7e is for PLA filament.

The device automatically records all information about properties it gathers from samples. Based on the tensile test results, the prepared filament's strength was compared to the most popular PLA filament. The results of the tensile test, including the ultimate tensile stress (MPa), tensile strength at failure, and maximum elongation for the prepared filament and the commercial PLA filament, are summarized in Table 5.

**Table 5:** Tensile properties of tested produced composite filaments.

Sample	Yield stress (MPa)	Elasticity modulus (MPa)	Stress at rupture (MPa)	Maximum elongation (mm)	Maximum strain (%)
rHDPE	6.92	715.47	14	112.51	22.3
rHDPE/rPET	7.76	588.46	11.61	16.89	18.42
15 % GF	7.47	527.3	12.24	13.4	19.78
30 % GF	8.98	761.96	12.05	8.2	21.19
PLA	11.45	1232.52	36.95	9.91	39.65

The mechanical characterization was carried out to compare the commercial PLA filaments. The result shows that 30 % GF-reinforced rHDPE and rPET have a larger yield stress when compared to rHDPE, rHDPE/rPET, and 15 % GF-reinforced rHDPE/rPET, which approximate similar to PLA larger yield stress's. However, in terms of elongation, rHDPE has a much higher value (112.5 mm) compared to PLA (9.91 mm), but with 30 % GF, the elongation is 8.2 mm, which is similar to PLA. Moreover, the filament fabricated from recycled plastic showed a small, varying diameter (1.75 ± 0.1 mm), creating stress concentration and yielding stress. The yield stress is expected to be much higher if a uniform-diameter filament is produced.

#### 3.1.4 Differential scanning calorimetry results

**Fig. 8:**
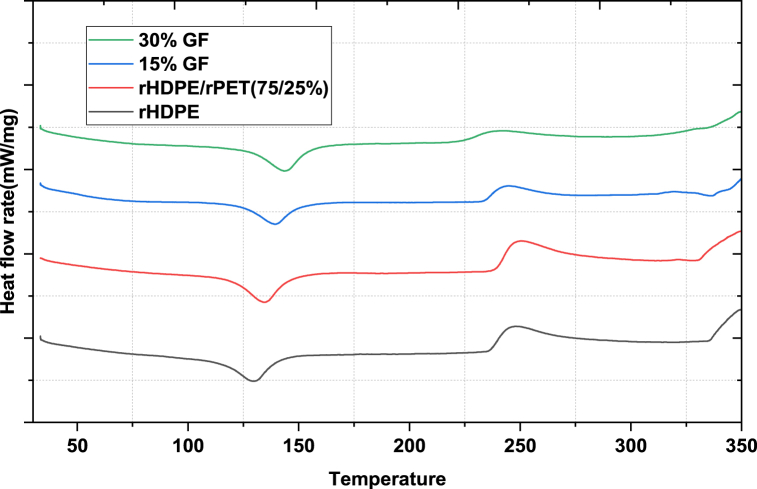
Differential scanning calorimetry first heating scan of filament.

A differential scanning calorimetry (DSC) study was used to examine the thermal stability or thermal behaviour of pure rHDPE, an rHDPE/rPET (75/25 wt%) blend, and 15 wt% and 30 wt% reinforced with glass fiber. The test can capture material transitions or phase changes as a function of temperature. Each filament's glass transition (*Tg*) and melting temperature (*Tm*) were shown using the DSC result curve, and the heat flow vs. temperature graph is displayed in Fig. 8.

The results of the first heating scan shown in Fig. 8 illustrate the thermal behaviour of processed composite filament and pure rHDPE after their respective thermal histories (glass transition temperature (*Tg*) and melting temperature (*Tm*)) which are reported in Table 4. The melting peak of pure recycled HDPE occurs around 129.5 °C, which is similar to the *Tg* of pure virgin HDPE in literature [31], which is claimed to be between 120 °C and 190 °C. Pure recycled HDPE compared with the polymer blends of rHDPE and rPET reinforced with 30 % glass fiber display a *Tm* moving towards higher temperatures, about 13 °C higher, whereas the thermal behaviour of polymer blends reinforced with 15 % glass fiber is 139.1 °C, which raises the temperature by about 9 °C. It is suggested that the composite filaments should be the same or similar to those of the rHDPE filaments, and they must be heated above thermal characteristics (*Tg* and *Tm*) temperatures. It can be used immediately in place of the base filament without requiring any changes to the printer's settings because it falls between the detected values for the pure polymer HDPE that have been previously reported [31].

The crystallinity value for melting (*Xm*) was calculated as Equation (3):

$$X_{c}\left(\%\right)=\frac{\Delta H_{m}}{f.\Delta H^{o}}\times 100\%$$

Where the melting enthalpy ($\Delta H^{o}$) of 100 % crystalline HDPE is taken as 293 J/g. [27], and $\Delta H_{m}$ was calculated from the graph from DSC by integrating the area of the reaction peak and the interpolated baseline between the beginning and end of the reaction using Equation (7).

(7) Heatingofmelting(ΔHm)=Areaunderthemeltingpeak(W.°Cg)Heatingrate(°Cs)

From the DSC result, the area under the peak of recycled HDPE can be calculated by using the origin lab software, and its value is 5.90944w.°Cg, and the Heating rate used here is 10°Cmin, if changed into (°Cs).

10°C⁄min=10°C⁄min×1/60°C⁄s=0.166667°C⁄s

Heatingofmelting(ΔHm)=5.90944w.°Cg0.166667°C⁄s=35.4566J/g

The crystallinity of melting ($X_{m}$) was:

$$X_{m}\left(\%\right)=\frac{35.4566\frac{J}{g}}{293\frac{J}{g}}\times 100\%=12.14\%$$

Similarly, the crystallinity of rHDPE, rHDPE/rPET (75/25), 15 % GF, and 30 % GF composite filaments are summarized in Table 6.

**Table 6:** DSC properties of produced composite filaments.

Sample	*T*_*g*_*(*^*o*^*c)*	*T*_*m*_*(*^*o*^*c)*	$\Delta H_{m}\left(\frac{J}{g}\right)$	*X*_*m*_(%)
rHDPE	98.4	129.8	35.4566	12.14
rHDPE/rPET (75/25 %)	114.9	135.8	40.9206	18.62
15 % GF	115.7	138.7	33.5489	19.08
30 % GF	124.8	142.1	38.6184	29.29

The thermal analysis of the filaments, or degree of crystallinity, increased with filler loading, which showed the thermal stability of the filaments increased.

### 3.2 3D printed sample characterization results

#### 3.2.1 Mechanical properties of printed tensile test result

**Fig. 9:**
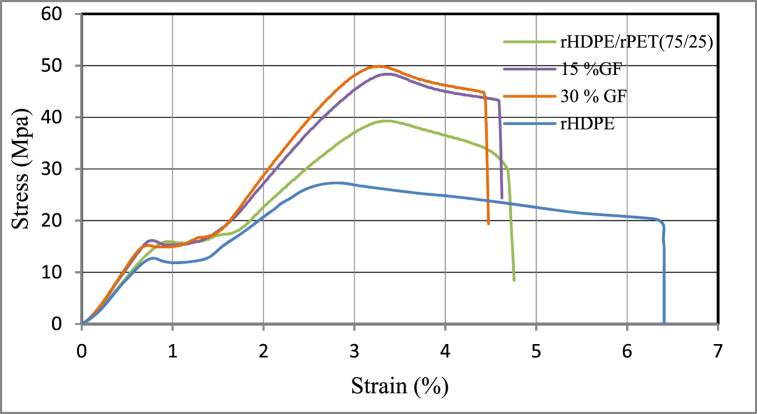
The tensile test result of the prepared 3D specimen.

The data recorded from the WP 310 universal testing machine and the stress-strain curves were displayed in Fig. 9.

It can be observed from Fig. 9 that an initial linear elastic behaviour up to yield strength around 16 MPa for 15 % and 30 % glass fiber reinforced rHDPE/rPET is followed by a non-linear region until the maximum stress is reached since it shows yield strength improvement compared to pure rHDPE, which is around 12 MPa. After the maximum stress peak, a reduction in stress with increasing strain was observed until failure occurred. Generally, a stress-strain curve represents the relationship between stress and strain. From the stress-strain curve, the following mechanical properties can be obtained: yield strength, ultimate tensile strength, Young's modulus, and elongation at break values.

**Fig. 10:**
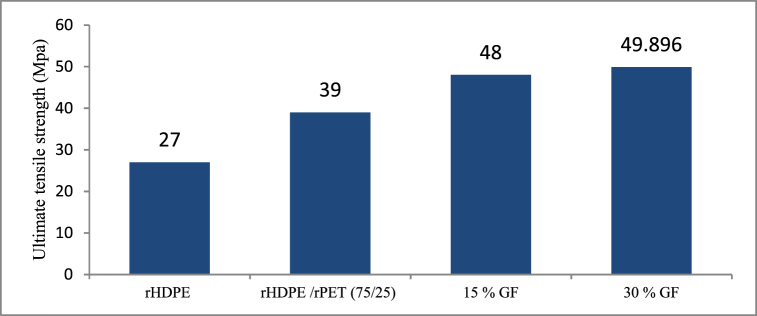
Variation of tensile strength with the addition of other materials.

Fig. 10 illustrates the ultimate tensile strength for recycled HDPE, rHDPE/rPET (75/25), and rHDPE/rPET blend composites as a function of short glass fiber content. The inclusion of glass fiber effectively increases ultimate strength. When each material is compared, there is an increase in ultimate tensile strength. In addition, it is found that the composite strength improves noticeably when materials are added. Compared to the rHDPE/rPET-15 wt% short glass fiber composites, the rHDPE/rPET-30 wt% short glass fiber composite has a higher tensile strength. To be precise, the addition of SGF has resulted in a significant increase in the UTS of the rHDPE/rPET matrix; this is primarily due to the reinforcing phase's primary role in carrying the majority of the load when subjected to external loading conditions. The strength of the composite material can be seen to be relatively high, and additive materials added to the rHDPE thermoplastic matrix would help to enhance the tensile strength. Again, as SGF content increased from 15% to 30%, the load-carrying capacity of the rHDPE/rPET composite increased, allowing the composite to carry more loads than the rHDPE/rPET/15 % SGF composite. One cannot simply state that raising the SGF concentration will increase UTS because more bonding and dispersion issues will occur. However, good inter-facial bonding and uniform SGF dispersion have been observed in the 3D-printed part because it shows higher tensile strength.

**Fig. 11:**
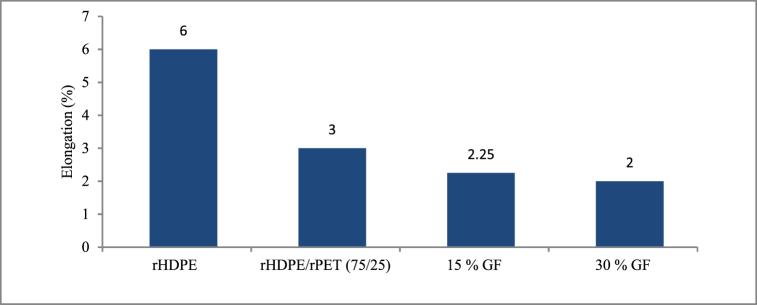
Variation of elongation with the addition of other materials.

When rPET was introduced to the rHDPE, the elongation percentage was reduced by 52 %, as shown in Fig. 11. Again, elongation further decreased due to the 30-weight-percent addition of SGF; with an increase in SGF, the polymer matrix produced improved results, although there was a decrease in elongation.

**Fig. 12:**
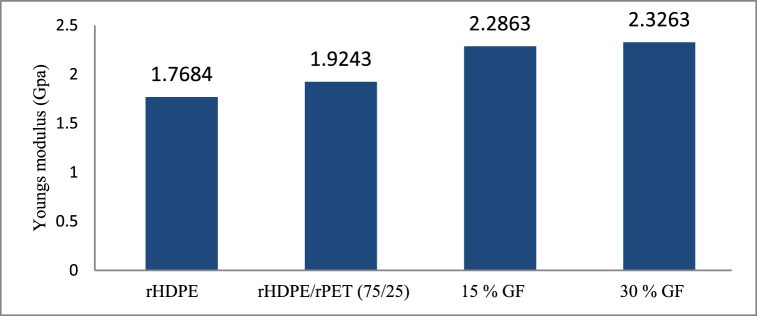
Variation of Young's modulus with short glass fiber content and materials.

Fig. 12 shows how Young's modulus of manufactured filaments varies due to the combination of rHDPE and rPET and the volume fraction of short glass fiber. As different materials are mixed, the composite filament of the 3D printed specimen's modulus also increases considerably. It is observed that as the fiber concentration increases, the composites' Young's modulus also increases; the fiber's orientation changes can thus be ignored because the fibers are aligned preferentially alongside the direction of molten filament flow in all the specimens. At low strain, the modulus is a material property that is highly delicate towards the fiber-matrix interface. The obtained values of 2326.3 MPa, 2286.3 MPa, 1924.3 MPa, and 1768.4 MPa for the young-modulus were for 30 % GF, 15 % GF, rHDPE/rPET (75/25), and pure rHDPE, respectively.

Moreover, mechanical characterization was performed to compare commercially available filament forms namely PLA (polylactic acid), ABS (acrylonitrile butadiene styrene), nylon, polypropylene (PP), polyethylene terephthalate (PET), and thermoplastic polyurethane (TPU). PLA is the widely used polymer filament materials and has an ultimate tensile strength of around 58 MPa, making it one of the more robust thermoplastics. On the other hand, it has an elongation to failure on the order of 2.02 % and an average modulus of elasticity of 3.3 GPa [32]**,** much lower than other thermoplastics such as ABS and nylon. Tensile strength values in the literature exhibit a range of 19–58 MPa. The peak stress value is between 34.6 and 35 MPa in the mechanical properties of FDM-produced ABS materials [33]. On average, ABS has a slightly lower tensile strength than PLA, with average tensile strengths of 28.5 MPa for ABS and 56.6 MPa for PLA, and average elastic moduli of 1807 MPa for ABS and 3368 MPa for PLA [34]. Table 7 shows the material's mechanical properties for the FDM-printed parts found in the literature review.

**Table 7:** FDM filaments reported in the literature and commercial filaments available in the market.

Polymer	Elastic modulus (GPa)	Ultimate tensile Strength (MPa)	Elongation at Break (%)	Reference
PLA	3.33	58.45	2.02	[35,36]
ABS	1.91	35, 30	6.2, 7.14	[37,38]
Nylon 66	1.74	39.9	19.3	[39]
PEETG	1.452	45	140	[40]
PP	1.25	35		[41]
TPU		46.2	694	[42]

The tensile strength, strain, and Young modulus results from this study were compared with different findings from the literature. Accordingly it was noted that 30 % GF-reinforced blends of rHDPE and rPET showed similar results as in other studies compared to PLA, PETG, and TPU with respect to tensile strength and were better than ABS, PP, and Nylon 66.

#### 3.2.2 Fractures specimen analysis

**Fig. 13:**
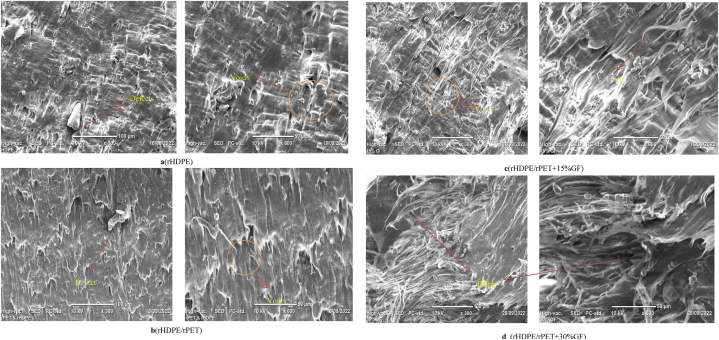
SEM results of manufactured filaments.

SEM was used to investigate the fractured surfaces of rHDPE, rHDPE/rPET blends, and glass fiber-reinforced composites that failed while testing the tensile. The microstructure of rHDPE, rHDPE/rPET, and its composites are shown in Fig. 13 (Fig. 13 (a) rHDPE, Fig. 13 (b) rHDPE/rPET (75/25) blend, Fig. 13c 15 % GF, and Fig. 13 (d) 30 % GF), which shows that short glass fibers are distributed uniformly throughout the matrix without clumping together. When two or more other materials are added, factors including reinforcing phase bonding and dispersion may affect load-carrying ability; however, the tensile test shows improved strength. The optimal blending conditions for screw extruders produced uniform distribution in the filament composites. The created filament used in FDM did not cause nozzle blockage even when it contained up to 30 % of the weight of glass fiber. Glass fiber dispersion was investigated in an rHDPE polymer matrix and revealed a uniform distribution of the fiber material without agglomeration.

As seen in Fig. 13, there is no glass fiber aggregation. Better properties are suggested, and it may be quantified that the surface-to-volume ratio is better. It is discovered that the GF is bound to the matrix. Additionally, because a polymer matrix's fibers are extruded alongside the matrix material, the fibers end up aligned with the direction of the nozzle's movement, which is why extrusion-induced shear stress led GF to align with the extrusion direction [43]. Improved mechanical characteristics were obtained due to the enhanced SGF content's failure to aggregate or harm the fiber surface after blending.

However, factors such as the reinforcing phase boundary and dispersion may affect load-carrying capability, and when injected into the rHDPE/rPET matrix, it is predicted to enhance the strength. When a 3D-printed part is subjected to external loading, the reinforcing phase carries most of the load. Blending in screw extruders under ideal manufacturing conditions resulted in uniform dispersion in 3D-printed composites. Using up to 30 % glass fiber by weight, the filament usage in FDM did not cause nozzle clogging. Some key elements, including extrusion temperature, compounding temperature, and preheating, enable a better-dispersed formation in a fiber-reinforced composite filament. Additionally, it has been discovered that increasing the filler's concentration from 15 to 30 wt percent can enhance its dispersion capabilities, resulting in better mechanical qualities.

## 4 Conclusion

3D printing using recycled plastics is an emerging technology that can be used to produce various models and has numerous advantages compared to traditional manufacturing processes. In this work, several composite 3D printing filaments were successfully extruded and tested for use in 3D printing applications. The filaments were produced from plastic waste and various weight fractions of glass fiber. Filaments with a 1.75-mm-diameter were made using pure rHDPE, rHDPE/rPET (75/25), rHDPE/rPET-reinforced SGF 15 %, and rHDPE/rPET-reinforced SGF 30 % by weight with the single-screw extrusion process. From the test, it was observed that SGF increased the samples' tensile strength. A decrease in deformation was observed with the incremental addition of SGF, indicating that the material was becoming brittle. The gradual addition of SGF improved dispersion capabilities, reduced voids, and prevented nozzle clogging.

This study concludes that there are a number of advantages to using plastic waste materials for different manufacturing purposes and proposes a method for developing novel materials for the FDM process. The current study can promote the sustainability of AM through life cycle assessments of waste plastic, material reuse by outlining a promising route for recycling manufactured plastic waste, and reducing environmental pollution through recycling and reusing as raw materials.

## Data availability

The research data are available from the authors up on request.

## CRediT authorship contribution statement

**Dame Ayane Tolcha:** Writing – original draft, Visualization, Validation, Methodology, Investigation, Formal analysis, Conceptualization. **Dereje Engida Woldemichael:** Writing – review & editing, Visualization, Validation, Supervision, Resources, Methodology, Investigation, Conceptualization.

## Declaration of competing interest

The authors declare that they have no known competing financial interests or personal relationships that could have appeared to influence the work reported in this paper.
